# Probing the Subcellular Localization of Hopanoid Lipids in Bacteria Using NanoSIMS

**DOI:** 10.1371/journal.pone.0084455

**Published:** 2014-01-07

**Authors:** David M. Doughty, Michael Dieterle, Alex L. Sessions, Woodward W. Fischer, Dianne K. Newman

**Affiliations:** 1 Division of Biology, California Institute of Technology, Pasadena, California, United States of America; 2 Division of Geological and Planetary Sciences, California Institute of Technology, Pasadena, California, United States of America; 3 Howard Hughes Medical Institute, Pasadena, California, United States of America; University of Groningen, Groningen Institute for Biomolecular Sciences and Biotechnology, Netherlands

## Abstract

The organization of lipids within biological membranes is poorly understood. Some studies have suggested lipids group into microdomains within cells, but the evidence remains controversial due to non-native imaging techniques. A recently developed NanoSIMS technique indicated that sphingolipids group into microdomains within membranes of human fibroblast cells. We extended this NanoSIMS approach to study the localization of hopanoid lipids in bacterial cells by developing a stable isotope labeling method to directly detect subcellular localization of specific lipids in bacteria with ca. 60 nm resolution. Because of the relatively small size of bacterial cells and the relative abundance of hopanoid lipids in membranes, we employed a primary ^2^H-label to maximize our limit of detection. This approach permitted the analysis of multiple stable isotope labels within the same sample, enabling visualization of subcellular lipid microdomains within different cell types using a secondary label to mark the growing end of the cell. Using this technique, we demonstrate subcellular localization of hopanoid lipids within alpha-proteobacterial and cyanobacterial cells. Further, we provide evidence of hopanoid lipid domains in between cells of the filamentous cyanobacterium *Nostoc punctiforme*. More broadly, our method provides a means to image lipid microdomains in a wide range of cell types and test hypotheses for their functions in membranes.

## Introduction

Biological membranes surround and demarcate all living cells. They consist of structurally diverse lipids and proteins thought to be arranged in chemically distinct microdomains [1]–[3] with proposed roles in macromolecule transport [2], signal transduction [2], and cell curvature [4]. A variety of methods are available to label membrane proteins without interfering with their cellular function [5]. In contrast, studying the behavior and localization of lipids into subcellular domains is challenging because current techniques permit observation of their behavior largely using fluorescent tags, either *in vivo* [6] or in synthetic lipid vesicles containing substantial amounts of detergent, fluorophore, etc.–conditions that can deviate markedly from native states and may be prone to artifacts [7].

Despite these challenges, studies of lipid subcellular organization have hinted at intriguing phenomena. For example, a liquid ordered phase was detected in model membrane vesicles based on both eukaryotic and bacterial cytoplasmic membranes and the bacterial outer membrane using a fluorescent dye [8]. In bacteria, the use of membrane dyes has suggested the presence of cardiolipin microdomains at the poles and septa of a variety of Gram-negative bacteria [9]–[11]; similarly, studies that utilized fluorescently-tagged lipids have shown that unsaturated lipids localize near the septum of *Shewanella livingstonensis* [6]. More recently, stable isotope labels and NanoSIMS have been use to track lipid localization and detect lipid rafts in model membranes [12] and in human fibroblast cells [13], [14]. Because it is unclear if lipid microdomains in occur in bacteria, and because the function of these microdomains remains controversial in any cell type [7], [13], [14], a major challenge is the development of approaches to examine membrane structure without perturbing it.

To overcome the limitations associated with non-native fluorophores and detergents, we developed a NanoSIMS-based stable isotope method to detect and image domains rich in hopanoid lipids in *Rhodopseudomonas palustris* TIE-1 and *Nostoc punctiforme* PCC73102. NanoSIMS has previously been used to detect labeled materials in *in vitro* model membrane systems [12], [15] and human fibroblast cells [13], [14] but isobaric interferences cause technical constraints that have limited its application to more highly labeled samples. To alleviate these constraints, we developed a different mass spectrometry approach based in simultaneous detection of ^1^H, ^2^H, ^12^C and ^13^C ions, which lowers the detection limit to one that permits bacterial lipid detection *in vivo* (see Methods). While the method we have developed has broad potential application, we focused on hopanoids because of our interest in their biological functions [16].

**Figure 1:**
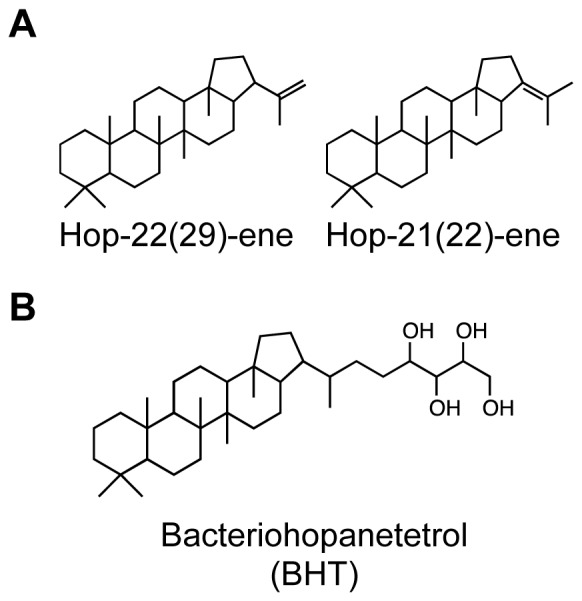
Chemical structures of hopanoid lipids used in this study. Structures of the two hopene isomers (A) and bacteriohopanetetrol (B) that were isotopically labeled with ^2^H, purified, and imaged in this study.

Hopanoids are bacterial pentacyclic triterpenoids (Figure 1 and Figure S1) that are structurally and biosynthetically similar to eukaryotic steroids [17]–[19] however their cellular roles are more poorly understood. In the past we have studied hopanoid localization in two different bacteria: *R. palustris*, an asymmetrically-dividing alpha-proteobacterium, where specific hopanoids differentially distribute between the cytoplasmic and outer membranes of mother and swarmer cell types [16] and *N. punctiforme*, a filamentous cyanobacterium, where hopanoids primarily localize to the outer membrane of its akinete cell-type [19]. In these previous studies, although we could localize hopanoids to particular membranes within specific cell types, we could not resolve their spatial distribution within these membranes [16], [19]. Nevertheless, this existing frame of reference made *R. palustris* and *N. punctiforme* attractive model systems in which to test the resolution and detection limits of our new method.

Stable isotopes provide a non-intrusive mechanism to label compounds, and are therefore much less likely to introduce artifacts than labeling approaches using other tags that alter the molecular structure of the lipids. Additionally, isotopic labels can readily be detected using mass spectrometry. In the case of NanoSIMS a primary Cs^+^ or O^−^ ion beam, as small as ca. 50 nm in diameter, is scanned across the surface of a sample. Secondary ions, generated by the primary beam, are analyzed by mass spectroscopy to create raster ion images of thin films and solids. Because NanoSIMS instruments employ multicollection, many different ions across a dynamic range of 19 mass units (for the 50 L) can be collected simultaneously, and isotope ratios can be measured with very high precision; moreover, maps of a given stable isotope ratio (for instance ^2^H/^1^H) can be used to visualize those regions of a sample linked to the addition of labeled substrates. We chose to label hopanoid lipids with deuterium (^2^H) because the natural abundance of ^2^H is very low (∼0.015%), providing a much lower minimal detection limit than for ^13^C, ^15^N, or ^18^O. This NanoSIMS method provides a means to advance our understanding of subcellular domains of lipids and proteins in diverse cell types.

## Results

### Isotope labeling and imaging of *R. palustris*

**Figure 2:**
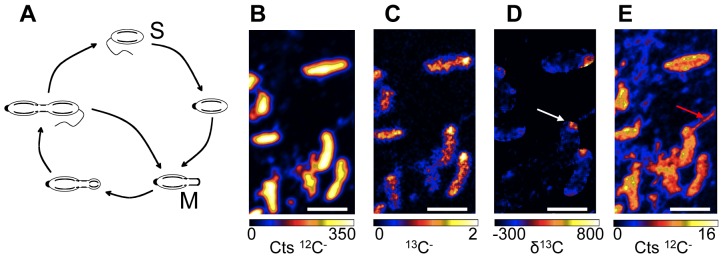
Cell biology of *R. palustris*. (A) Cell cycle of the budding mode of cell division displayed by *R. palustris* in which motile swarmer cells (S) lose their flagellum and develop into tubed mother cells (M) that give rise to new swarmer cells. (B–E) A pulse of ^13^C labeled acetate added 30 min prior to the fixation of the cells allows for the visualization of the *R. palu*stris cell cycle. Quantitative raster ion images of ^12^C^−^ (B) and ^13^C^−^ (C) were used to calculate the δ^13^C image (D) that indicates the presence of ^13^C enrichment at one pole of the cells as indicated by the white arrow. (E) A bacterial flagellum is visible, indicated by the red arrow, within an early raster frame in the ^12^C^−^ ion image confirming that the ^13^C is preferentially associated with the growing bud. Scale bars are 2 µm. Images shown are representative of 3 fields of view and 3 independent biological experiments.

We focused first on *R. palustris* because it produces many different hopanoids and has a well-defined cell cycle [16], [20]. Because cholesterol is structurally similar to hopanoids and is readily detectable by the fluorescent dye filipin [21], we used cholesterol as a control to test whether *R. palustris* could take up hydrophobic isoprenoid compounds (Figure S4). Moreover, using previously constructed hopanoid-deficient *R. palustris* mutants [22], we could perform complementation experiments to determine whether the addition of exogenous labeled hopanoids restored a hopanoid-dependent phenotype; this control enabled us to assess whether any observed localization pattern was biologically significant. We constructed mutant strains of *R. palustris* in which the outer membrane associated protein Pal [23] was fused to the fluorescent protein mCherry. The fluorescent construct was visible in the wild type hopanoid background, however, it was much less intense in the mutant strain that does not make hopanoids. Upon the addition of either hopene or BHT, mCherry fluorescence increased (Figure S5). Although it is unclear why fluorescently tagged Pal is muted in the absence of hopanoids, our observations show that exogenously added hopanoids can restore bright fluorescence to the mutant strain. Because *R. palustris* divides asymmetrically by budding from one pole (Figure 2A), we reasoned that we could mark the growing bud with a ^13^C label supplied with the carbon source used for growth and image this ‘growth’ label concomitantly with other ions. When we incubated cells with ^13^C-acetate for 30 min prior to harvesting for NanoSIMS analysis, the ^13^C incorporation was more intense at one pole of the cell (Figure 2B, C, D). The observation of flagella attached to this end, using a 1.8 pA primary ion beam (Figure 2E), confirms that this was the growing pole. Previous work established that flagella are associated with the budding, or “swarmer” cell and not the mother cell [20].

**Figure 3:**
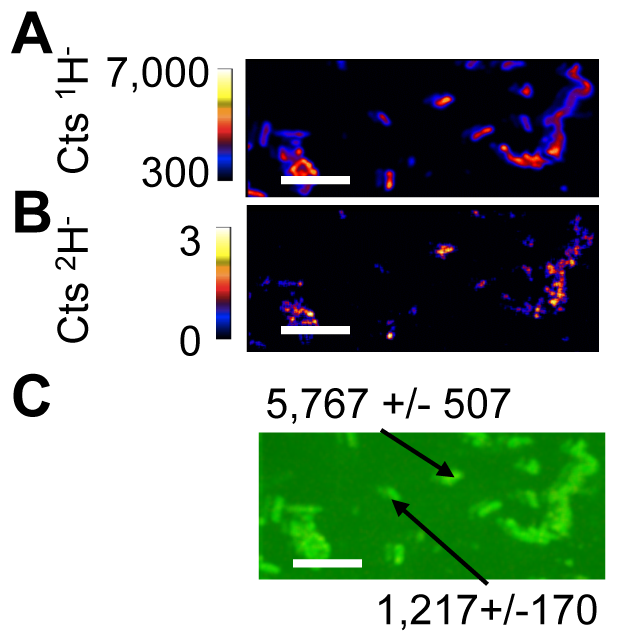
Detection of exogenously added ^2^H-labeled BHT in *R*. *palustris*. NanoSIMS images showing the count rates of ^1^H^−^ (A) and ^2^H^−^ (B) ions. Note that the ^2^H abundance is patchy within cells. (C) A non-quantitative overlay showing ^2^H^−^ on top of ^12^C^−^ counts to mark cells. δ^2^H values for regions of interest encompassing two different cells marked by arrows. Cells were defined by contiguous regions generating a ^12^C^−^ count rate greater than 300 cts s^−1^ (shown as bright in the green image) and had a bulk δ^2^H of 638±10, indicating the broad incorporation of labeled hopanoids. The reported error reflects the standard error of total ion counts within each of the indicated cells. Images shown are representative of 20 fields of view and 3 independent biological experiments. All scale bars are 5 µm.

**Figure 4:**
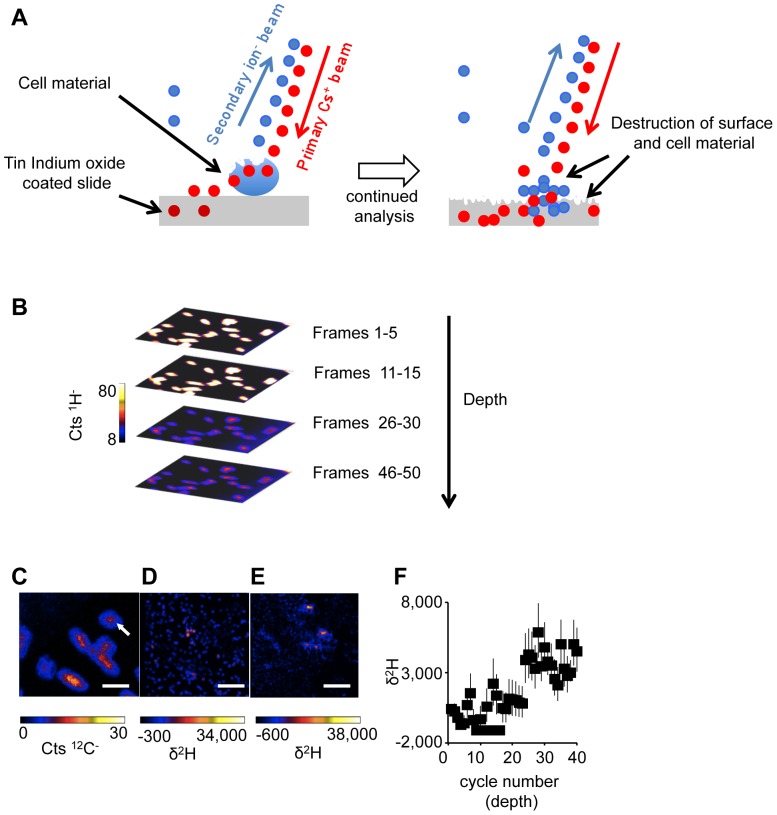
Depth profiles of *R. palustris* cells using NanoSIMS. (A) Conceptual illustration of NanoSIMS analysis of a cell. As the beam is rastered over the surface of the sample in many cycles, more material is removed from the sample surface and thus time (i.e. cycle number) relates to depth. (B) A stack of ion images binned by cycle number shows how cells are degraded over time by the ion beam. Quantitative images of ^12^C^−^ (C), ^2^H^−^ frames 5 to 10 (D), and ^2^H^−^ frames 30–35 (E) are shown to illustrate changes that occur in the ^2^H^−^-labeling patterns as the primary beam burns through the cells. (F) A depth profile of the cell indicated by the white arrow in panel C provides a quantitative example of a cell initially appearing unlabeled but becoming positive for the label as the opposite side of the cell is sampled. All scale bars are 2 µm. Error bars reflect the standard error of the total number of ions. Images shown are representative of 5 fields of view and 3 independent biological experiments.

^2^H-labeled hopanoids were purified and added to the medium as described in the Methods (and see Figures S1, S2 and S3). Next we tested the ability of *R. palustris* to take up exogenously supplied ^2^H labeled bacteriohopanetetrol (BHT) or hopenes. Cells from each image were analyzed using the L'IMAGE PV-WAVE software package (written by Larry R. Nittler) and the average δ^2^H values (see supplementary information) for BHT and hopene treated cells were 196.6+/−9.7‰ and 2,244.0+/−20.8‰, respectively. These values were significantly enriched above unlabeled material that had a background δ^2^H value of −237+/−167‰, indicating the uptake of labeled hopanoids. In NanoSIMS imaging it is common to bin pixels together into independently selected regions of interest (ROI) to attain better statistics on isotope ratios. Individual cells had different isotope ratios, for example in the BHT treated cells in Figure 3, one cell was labeled with a δ^2^H of 5,617+/−507‰ while a nearby cell shows only a δ^2^H of 1,217+/−170‰ (Figure 3). In part the discrepancy between label incorporation can be attributed to subcellular heterogeneity in both BHT and hopene treatments. For example, cells often appeared not to contain any label, however, when repeatedly analyzed for an extended period of time discrete regions of cells became significantly ^2^H-enriched as the primary ion beam ablated material and began to sample the opposite side of the cell (Figure 4). These data are consistent with the notion that hopanoid lipids are concentrated in patches within membranes. Although we were able to distinguish cellular orientation using the ^13^C label and detect deuterated hopanoid lipids, we were unable to detect significant trends for the subcellular localization of hopanoids in *R. palustris*. In part, our inability to easily distinguish repeatable patterns was due to the extremely small cell size of *R. palustris* (typically less than 1 µm in length and ∼500 nm in width), which both challenged the resolution limits of the NanoSIMS and also limited the yield of ions that could be collected before the cell material was obliterated. Nevertheless, *R. palustris* was a good system in which to begin our study of exogenous hopanoid additions due to the availability of mutant strains for complementation. To overcome the size limitation, we repeated our experiments with the larger filamentous cyanobacterium, *N. punctiforme*.

### Isotope labeling and imaging of *N. punctiforme*

*N. punctiforme* forms multicellular filaments that can be hundreds of cells in length, with each cell typically about 4 µm long and 4 µm wide. Although cell material shrinks following dehydration, cells were sufficiently large for NanoSIMS studies. *N. punctiforme* produces three distinct cell types. Vegetative cells perform oxygenic photosynthesis and fixation of CO_2_. Heterocysts form in response to fixed nitrogen starvation and are dedicated to the metabolic task of fixing N_2_, which is then transported to the vegetative cells. During phosphate or energy starvation, *N. punctiforme* also produces a spore-like cell type, termed an akinete, which serves as a protective structure against cold and dehydration. Akinetes are found along filaments approximately at the midpoint between heterocysts and are distinguished by their increased size relative to vegetative cells [24].

**Figure 5:**
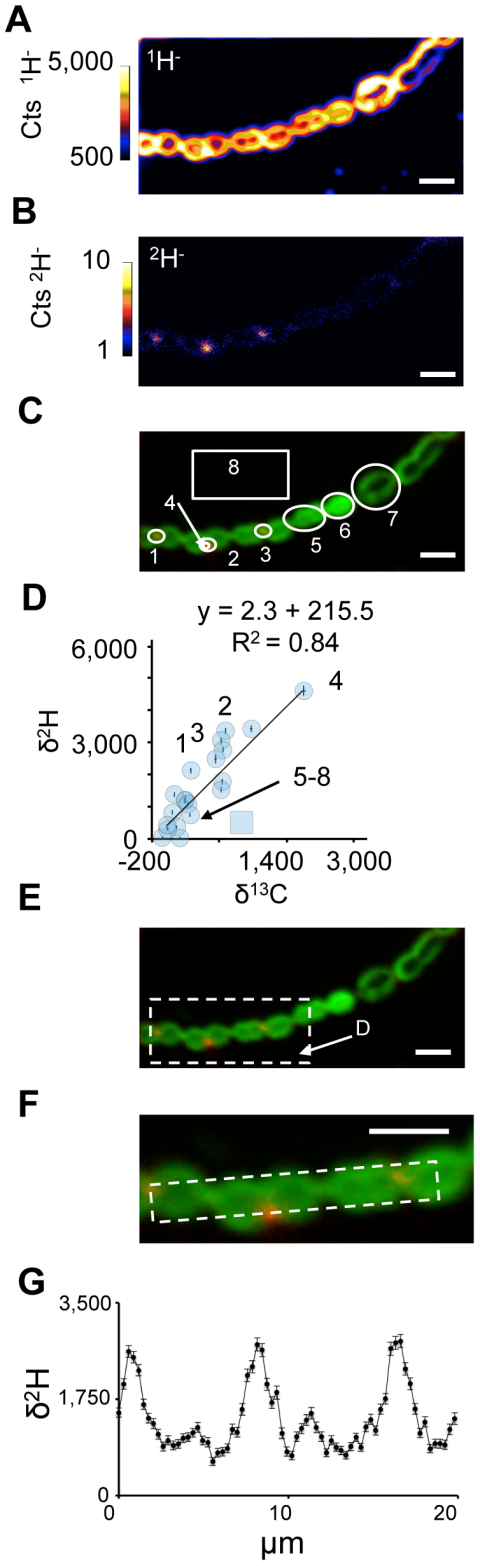
Analysis of BHT localization in *N. punctiforme* cells. NanoSIMS images of ^1^H^−^ (A) and ^2^H^−^ (B) ion count rate and a non-quantitative overlay (C) of the raster images depicting their relative localizations with ^1^H^−^ ions shown in green and ^2^H^−^ ions shown in red. The thin white circles in C denote the regions of interest (numbered 1–8) selected for the pooled analysis of data in panel D. The white arrow indicates that region of interest 4 is within region of interest 2. (D) Plot depicting the δ^13^C and δ^2^H values for the regions of interest (numbered blue circles) and all image pixels containing greater than 100 cts s^−1^ ^1^H^−^ ions, which encompasses all biomass in the image (blue square). Additional regions of interest points shown as are data taken from other ion images (unnumbered blue circles). The line is an ordinary least squares regression of interest shown in C. (E) A white dashed line indicates the region enlarged in panel F. (F) The white dashed line indicates a region of interest along a filament for the isotopic profile shown in G. (G) δ^2^H values along the filament highlight a periodicity with labeled BHT concentrated at the junctions between cells. All scale bars are 5 µm. Error bars in (G) reflect the standard error of the total number of ions. Images shown are representative of 5 fields of view and 3 independent biological experiments.

Previously, we determined that hopanoids were preferentially concentrated in the outer membranes of akinete cell types, but traces were also seen in membranes surrounding vegetative cells and heterocysts [16]. To determine whether more subtle subcellular localization patterns existed, we added ^2^H-labeled BHT or hopenes to actively growing cultures of *N. punctiforme* (Figure 5). Following a 3 day exposure to ^2^H-labeled BHT, cultures were supplied with 2 mM NaH^13^CO_3_ buffered to pH 7 with 10 mM MOPS and allowed to incubate an additional 4 hours, then fixed and prepared as described for *R. plaustris*. ^2^H-enrichment was clearly visible in *N. punctiforme* and appeared in patches in between vegetative cells (Figure 5A–C). In Figure 5D δ^2^H is plotted against δ^13^C for all regions of interest. These data indicate that bulk cell material, defined as pixels exceeding 300 counts of ^12^C, is not strongly labeled with either hopanoid or ^13^C. In contrast, there was a positive correlation between the BHT, δ^2^H, label and δ^13^C (Figure 5D). The BHT label was enriched in patches between cells and when we binned the pixels to generate profiles along the filament's center the rest of the cell material rarely exceeded a δ^2^H of 1,000‰ (Figure 5E–G).

**Figure 6:**
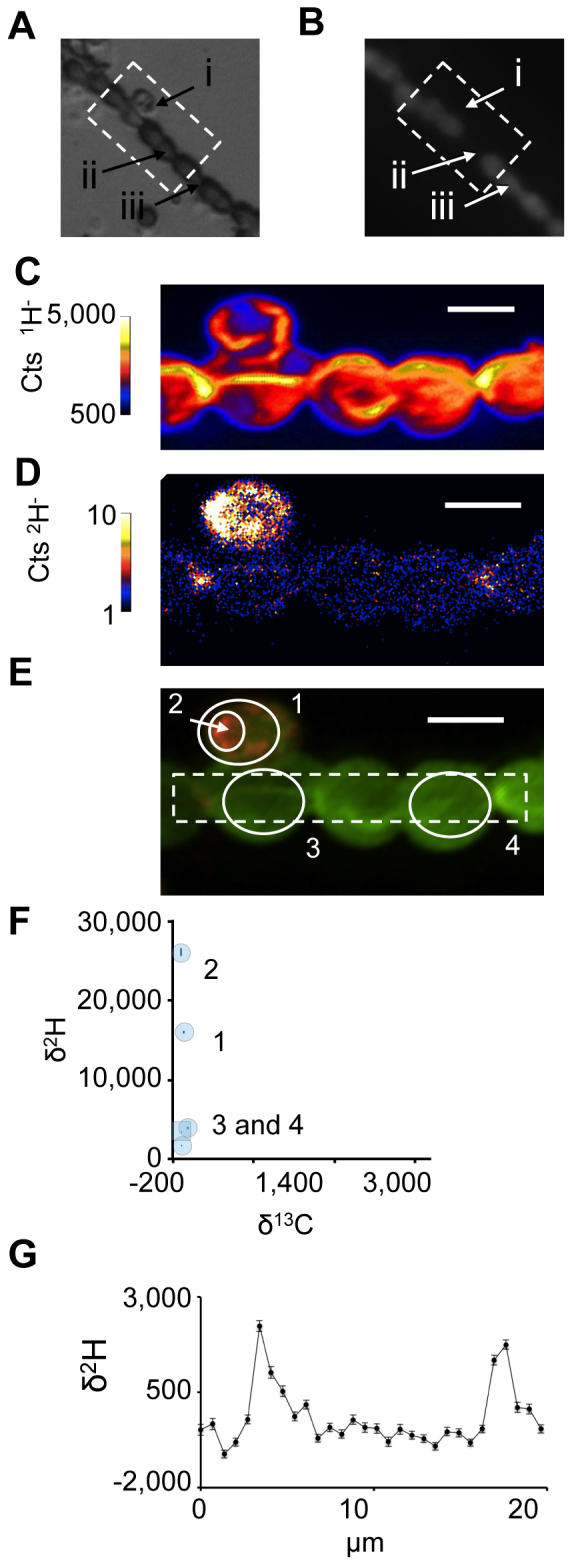
Analysis of hopene localization in *N. punctiforme* cells. (A) Phase contrast and (B) fluorescence images indicating the presence of a jettisoned akinete envelope (i), a heterocyst (ii), and vegetative cells (iii). NanoSIMS images of ^1^H^−^ (C) and ^2^H^−^ (D) ion count rates and a non-quantitative overlay of the two ions depicting their relative localizations (E) with ^1^H^−^ ions shown in green and ^2^H^−^ ions shown in red. Note the substantial concentrations of labeled BHT within the akinete envelope. (F) δ^13^C and δ^2^H values for the regions of interest denoted by solid white lines in E (numbered blue circles) and all image pixels containing greater than 100 ^1^H^−^ ions (blue square). The dashed white lines in E depict the region selected for the isotopic profile shown in (G). A plot of δ^2^H values along the filament indicates the BHT label is localized near cell junctions. All scale bars are 5 µm. Error bars reflect the standard error of the total number of ions in the selected region. Images are representative of 5 fields of view and 3 independent biological experiments.

Next we expanded our experiments to include the detection of ^2^H-labeled hopene in *N. punctiforme*. Bulk cell material treated with ^2^H-labeled hopene had a δ^2^H of 2,234+/−20.8 ‰, which is significantly enriched above background. Again the ^2^H label appeared in patches with similar δ^2^H values to those observed in BHT labeled cells, however, unlike BHT the hopenes did not positively correlate to the ^13^C label (Figure 6). These data suggest that although BHT and hopene both localize between cells of *N. punctiforme* BHT, they have different patterns of localization. Interestingly, some of the cells of *N. punctiforme* had shed akinete envelopes, a phenomenon associated with improved nutrient conditions likely associated with the addition of the hopene-containing medium. Akinete envelopes had δ^2^H values as high as 30,000‰ (Figure 6F). While the enrichment of hopanoids in akinete envelopes is consistent with our previous work [16], to determine whether the subcellular localization patterns observed here are biologically significant will require complementation experiments similar to those performed with *R. palustris*. Hopanoid-deficient mutant strains of *N. punctiforme* are in construction for these experiments.

## Discussion

Diverse techniques in cell biology have long allowed researchers to infer differences in intercellular distribution of lipids. For example, differences in the lipid profiles of mother and swarmer cells of *R. palustris* and the high cardiolipin and phosphatidic acid content of minicells of *Escherichia coli* have suggested that some lipids may have specific subcellular localization [10], [19], [25]. Our stable isotope labeling and imaging method allows a direct test of such hypotheses. We applied it to study the intra- and intercellular localization of hopanoids in two different types of bacteria. The different localization patterns we observed for hopene and BHT in *N. punctiforme* suggest that different hopanoid structures have specific and potentially diverse cellular functions. It is noteworthy that hopanoids localize to regions of high curvature in *N. punctiforme* (i.e. between cells in *N. puntiforme* filaments). These observations are consistent with our previous observation that BHT production in *N. punctiforme* coincides with the appearance of akinete cells that are associated with the break up of the cellular filament [16], [24]. Future imaging and *in vitro* experiments can test the hypothesis that BHT may help generate cell curvature, as has been demonstrated for certain types of cholesterol in synthetic membranes [26].

More broadly, the general applicability of this method has the potential to advance studies of diverse lipids in many cell types [13]–[15]. Nevertheless, important technical challenges remain. Our current technique of sample preparation relies on fixation with paraformaldehyde followed by dehydration in ethanol. We acknowledge the possibility that this method of sample preparation could have allowed hopanoid lipids to change in their localization during the fixation procedure. Future efforts to integrate cryo preparation and transfer into the NanoSIMS would have great value for improving this approach.

In summary, NanoSIMS is a versatile technology and other isotope labels that could be incorporated into membrane compounds (e.g. ^13^C, ^15^N, and ^34^S) could be readily included in future imaging experiments. Isotopic-labeling of membrane compounds could be expanded to include radioisotopes such as ^3^H that would provide a greatly enhanced limit of detection due to the effective absence of background abundance of ^3^H and a lack of interfering species. Numerous opportunities exist for creative expansions of this method to address longstanding questions regarding the behavior and organization of lipids in microdomains in a wide range of bacterial and eukaryotic cells.

## Methods

### Cell culture conditions for *R. palustis* and *N. punctiforme*

Wild type, *ΔhpnH*, and *ΔhpnPΔhpnO, glmX*::P_lacZ_–Pal–mCherry, *Δshc glmX*::P_lacZ_–Pal–mCherry *R. palustris* strains were cultured in medium consisting of 3 g/L yeast extract, 3 g/L peptone and buffered with 10 mM MOPS pH 7 (YP medium). For cultures grown in the presence of ^2^H_2_O an additional 5 mM disodium succinate was added and 100 µm isopropyl β-D-1-thiogalactopyranoside was added to the medium to induce the expression of mCherry fusion proteins. All cultures were maintained at 30**°**C on an orbital shaker at 250 rpm.

Vegetative cultures of *N. punctiforme* were maintained in Allen and Arnon Medium (AA medium) [24] except KNO_3_ was not added as a N-source to promote the formation of heterocycts. Cultures were maintained at 30**°**C and continuously illuminated by fluorescent light.

### Purification of ^2^H-labeled hopanoids

We first constructed *R. palustris* mutant strains to facilitate the purification of specific hopanoids. The *hpnH* gene is essential to the production of all extended hopanoids, and its deletion produced a mutant strain, *ΔhpnH*, that accumulates the C_30_ hopene isomers, diplopterol, and tetrahymanol (Figure S1). By deleting both the *hpnP* gene, responsible for producing 2-methylhopanoids [27], and the *hpnO* gene, essential for the production of bacteriohopaneaminotriol [18], we generated a double mutant (*ΔhpnPΔhpnO*) that accumulates bacteriohopanetetrol (BHT) (Figure S1). The *ΔhpnH* and *ΔhpnPΔhpnO* mutant strains were gradually adapted to grow in medium consisting of 70% ^2^H_2_O by sequential passage of the culture in medium containing 0, 21, 28, 35, 42, 49, 56, 63, and 70% ^2^H_2_O. Following growth in 70% ^2^H_2_O, cultures were harvested and lipids extracted using a modified Bligh and Dyer procedure [28] with dichloromethane (DCM) replacing chloroform. Total lipid extracts (TLE) were separated by chromatography on a silica gel column. Six fractions were eluted with two column volumes of each of the following solvents: F1 hexane; F2 hexane: DCM (4∶1); F3 DCM; F4 DCM: ethyl acetate (4∶1); F5 ethyl acetate; F6 ethyl acatate: methanol (4∶1); F7 methanol [24]. The hydrocarbon fraction of the TLE from the *ΔhpnH* mutant consisted of a mixture of hop-22(29)-ene and hop-21(22)-ene (Figure S2A, B). Fraction 7 from the *ΔhpnP ΔhpnO* double mutant contained BHT (Figure S3B, C). To ensure purification of BHT and to remove the potentially exchangeable ^2^H on the polyhydroxylated head group, fraction 7 was derivatized as the acetate esters with acetic anhydride and pyridine, and loaded onto a second silica gel column. Acetylated BHT was eluted in fraction 3 and the acetate ester was removed under basic hydrolysis to return BHT to its underivatized state. These fractions contain purified lipids with hopanoid backbones with a non-specific ∼40% ^2^H label. Because ^2^H label was bound to C atoms and aliphatic C-bound H is not interchangeable with solvent on laboratory time scales [29], the labeled hopanoids could be stored at 4**°**C for later use.

### Introducing hopanoid label to the medium

To test the ability of isoprenoid-like compounds to enter aqueous medium and be taken up by *R. palustris*, we designed a fluorescence-based technique using filipin, a sterol specific dye [21]. Cholesterol was dissolved in methanol and a 0.2 µg aliquots of cholesterol were placed into glass vials and allowed to dry. 200 µl YP medium was pipetted into the vial and allowed incubate for 12 hours at 30**°**C. The YP medium with cholesterol was then used to resuspend wild type cells, the cells were placed in a fresh vial, and allowed to incubate for 30 min. Cells were harvested by centrifugation, washed, treated with filipin for 5 min, washed again and visualized by phase contrast and fluorescence microscopy. Filipin stained *R. palustris* cells treated with cholesterol containing medium Fig. S4A, B), suggesting that cholesterol, an isoprenoid with structural similarity to hopanoids, is at least sparingly soluble in the medium and can be transferred to cells. As a control we repeated the experiment but did not add cholesterol to the methanol. When *R. palustris* cells that were not exposed to cholesterol were exposed to filipin and then washed, cells did not become fluorescent indicating that the dye did not detect hopanoid lipids and was specific to sterols (Figure S4C, D).

### Construction and complementation of strains expressing a mCherry fusion of Pal

Previous research on *C. crescentus* utilized the protein Pal to mark the cellular division site [23]. *Because C.* crescentus *and R. palustris* are both alpha-proteobacteria, we reasoned that Pal could serve as a marker of the cell cycle in our studies. As hoped, our fluorescently-labeled Pal construct was visible in wild type *R. palustris*, with a localization pattern similar to that observed in *C. crescentus*; intriguingly, it fluoresced poorly in a mutant strain (*Δshc*) that lacked hopanoids (Figure S5). We thus utilized this mutant to test the ability of exogenously added hopanoids to restore (or “complement”) the wild type Pal localization phenotype. Table S1 contains all strains, plasmids and primers used for the construction of the mCherry fusion protein and transformation was conducted as described previously [22].

When strain *Δshc glmX*::P_lacZ_–Pal–mCherry was supplied with 1 µg/ml of hopene fluorescence increased within 30 min and resembled the localization patterns observed in the hopanoid producing stain, *glmX*::P_lacZ_–Pal–mCherry (Figure S5E, F). When *Δshc glmX*::P_lacZ_–Pal–mCherry was supplied with1 µg/ml of BHT, fluorescence increased within 30 min, but localization patterns observed in the wild-type were not clearly observed (Figure S5G, H). These experiments suggest ^2^H-labeled hopanoids are able to complement hopanoid associated phenotypes.

### Preparing cells for NanoSIMS analysis

Hopenes or BHT were suspended in DCM, and 0.2 µg aliquots were transferred into separate glass vials and allowed to dry. 50 µl of YP medium or AA medium was then added to each vial and allowed to incubate 12 hours at 30**°**C. *R. palustris* and *N. punctiforme* were harvested by micro-centrifugation at 14,000 rpm for 1 min, resuspended in the hopene or BHT containing medium, placed in a new flask, and then allowed to incubate for the indicated length of time. Cells were fixed with the addition of paraformaldehyde (1% final concentration), washed 3 times in water, and dehydrated by subsequent 1 min washes in 25, 50, 75 and 100% ethanol. 0.5 µl of cell suspended in ethanol were then spotted on a conducting indium tin oxide (ITO) coated glass slide, to ensure a conducting surface, and imaged via NanoSIMS.

### NanoSIMS protocol for the detection of ^1^H^−^, ^2^H^−^, ^12^C^−^, and ^13^C^−^

To demonstrate the feasibility and accuracy of our technique to measure ^2^H-enrichments within cells, whole cells grown in the presence of 0, 10, 35 and 70% ^2^H_2_O were analyzed by NanoSIMS. Prior work [9] measuring ^2^H/^1^H in synthetic lipid bilayer membranes used a Cameca NanoSIMS 50 to detect ^2^H-enrichments by analyzing the relative abundances of ^12^C^2^H^−^ vs. ^12^CH^−^ secondary ions with a stated mass resolving power of ∼6,800. In our initial experiments on the Cameca NanoSIMS 50 L (a similar instrument but with seven instead of five detectors), we found that the same approach is insufficient to resolve key isobaric interferences from ^12^C^2^H^−^ at any mass resolving power (MRP) achievable by this instrument (Figure S6) unless the samples of interest contain percent levels of ^2^H^−^, an unattractive criterion for stable isotope probes within cells. We therefore decided to measure ^2^H/^1^H from the ^1^H^−^ and ^2^H^−^ ions directly. The large dynamic range of the NanoSIMS 50 L allowed us to collect ions of mass 1 and 13 Da concurrently on different electron multiplier detectors, so we could produce δ^2^H and δ^13^C images of the same cells from a single analysis. Working at a MRP of ∼3,000 is sufficient to separate both ^1^H^−^ from ^2^H^−^, and ^13^C^−^ from ^12^CH^−^, while affording higher transmission and ion count rates. The uncertainties of our isotope ratio measurements were assessed from ion images of many cells grown in known concentrations of ^2^H, and are approximately 10% (Figure S7). The precision associated with the measurements is controlled largely by Poisson counting statistics (and thus the size of areas integrated in regions of interest) and was typically 100‰ and 5‰ (1σ) for δ^2^H and δ^13^C, respectively, for regions 25×25 µm in size and pixels yielding at least 300 counts of ^12^C^−^. When all the data from a picture of unlabeled cells is pooled together, the background ratio of ^2^H/^1^H was 0.00016 corresponding to a δ^2^H value near zero. Stable isotope ratios remained linear across a concentration range from natural abundance (0.0156%) to 70% ^2^H-labeled cell materials (Figure S7).

### Data collection and image processing

Because NanoSIMS is a destructive analytical technique and cells provide a finite amount of material, we optimized our parameters to maximize ion count rates per cell, while maintaining a small high-resolution primary ion beam. As with any imaging technique NanoSIMS has the ability to use empty resolution in which pixel size is smaller than the beam size. We found empty resolution to be too consumptive of cell material, particularly in *R. palustris*. By calculating pixel size we were able to maintain pixel sizes of 60 nm^2^, thus eliminating empty resolution. Following NanoSIMS analysis samples were visualized by SEM to evaluate the sputtering depth. *R. palustris* cells were completely sampled and removed from the imaged regions of the slide, consistent with the disappearance of ^12^C after several frames of analysis disappearance during NanoSIMS analysis. In contrast, *N. punctiforme* cells remained intact, suggesting a small sampling depth limited largely to material derived from the cell envelope (<2 nm)–consistent with the expected lifetime of cellular materials given our operating conditions (Figure S8; [30]).

### Image processing and statistics

With the goal of quantifying ^2^H^−^ we used L'image software, a versatile software program designed to render ion images and analyze NanoSIMS data. Using a dead time correction of 44 ns we rendered images in two steps. First, in the event that multiple images where taken of the same cell material L'image is able to align the panes creating image stacks. Second, we produced δ^2^H and δ^13^C by integrating the total counts of either ^1^H^−^ and ^2^H^−^ or ^12^C^−^ and ^13^C^−^ raster images (equations provided in Figure S7). The statistical analysis of the L'image software package, based upon the standard error of Poisson counting statistics, is reported for all regions of interest and profiles.

## Supporting Information

Figure S1 **The pathway of hopanoid biosynthesis leading from squalene to the production of hopanoids.** Colored X's indicate the disruption of hopanoid biosynthesis through the deletion of known biosynthetic genes. Genes were deleted to control hopanoid production of specific hopanoid lipids and enable the production of unique labeled compounds. Shown in red is the HpnH protein essential for the production of adenosylhopane from Hop-22(29)-ene. Shown in blue are deletions of HpnO, the gene responsible for the production of bacteriohopaneaminotriol, and HpnP, the gene essential to the production of 2-methylbacteriohopanetetrol.
(PDF)

Figure S2 **Purification of ^2^H-labeled BHT.** (A) GC/MS chromatogram of total lipid extract of wild-type unlabeled *R. palustris* shows a range of hopanoid products. (B) GC/MS chromatogram of pure unlabeled BHT. (C) Comparison of the mass spectrums of unlabeled (top) and ^2^H-labeled (bottom) BHT. The shift of the 191 peak to approximately 202 is consistent with about a 48% non-specific ^2^H label.
(PDF)

Figure S3 **Purification of ^2^H-labeled hopenes.** (A) GC/MS chromatogram of purified hopene isomers. (B) Mass spectrum of unlabeled hopene (top) compared to the ^2^H-labeled spectrum (bottom). The shift of the 191 peak to approximately 200 is consistent with about a 39% non-specific label.
(PDF)

Figure S4 **Filipin, a cholesterol specific dye** [**21**] **, was used to determine the ability of** ***R. palustris*** **to uptake exogenously added cholesterol.** (A) Phase contrast and (B) fluorescence images of *R. palustris*, stained with filipin, following exposure to cholesterol containing medium. (C) Phase contrast and (D) fluorescence images of *R. paustris* exposed to medium that did not contain cholesterol. All scale bars are 5 µM. Images are typical of 3 fields of view and 3 biological replicates.
(PDF)

Figure S5 **Localization and fluorescence of mCherry labeled Pal in** ***R. palustris*** **.** (A) Phase contrast and (B) fluorescence images of the hopanoid-producing *R. palustris* strain *glmX*::P_lacZ_–Pal–mCherry fluoresce following induction by IPTG. (C) Phase contrast and (D) fluorescence images of mCherry Pal in the hopanoid negative mutant *Δshc glmX*::P_lacZ_–Pal–mCherry mutant show reduced fluorescence. (E) Phase contrast and (F) fluorescence images show the complementation of the *Δshc glmX*::P_lacZ_–Pal–mCherry phenotype 30 min after the the addition of 1 µg/ml of exogenously added hopenes. Yellow arrows in e indicate the presence of small vesicles formed after the addition of the hopene. (G) Phase contrast and (H) fluorescence images showing the complementation of the fluorescent signal of the *Δshc glmX*::P_lacZ_–Pal–mCherry phenotype 30 min after the the addition of 1 µg/ml of exogenously added BHT. Cells shown in the insets are indicated by the red arrows. All cultures were induced for 8 hours with 100 µm IPTG. All scale bars are 5 µm. Images are typical of 4 fields of view and 3 biological replicates.
(PDF)

Figure S6 **Comparison of methods used for the detection of ^2^H/^1^H.** Prior methods to measure ^2^H/^1^H relied on measurements of the ^12^C^2^H^−^ and ^12^CH^−^ ions [15], the former of which is subject to an isobaric interference from ^12^CH_2_^−^ that precludes this analytical set up for imaging labeled lipids within cells at any reasonable mass resolving power (MRP). (A) Mass spectrum ca. 14 A.M.U. of natural abundance *R. palustris* cells at the MRP used by Kraft *et al*. [15]. (B) Same mass spectrum with the highest MRP available at those conditions. Instead the NanoSIMS 50 L enables detection of ^1^H^−^, ^2^H^−^, ^12^C^−^, and ^13^C^−^ concurrently in the same analysis. Direct comparison of ^2^H^12^C^−^ (C) and ^2^H^−^ (D) yields from the hopene labeled cell material (also shown in Fig. 6). The images in c and d are representative of 5 fields of view and three biological replicates. Scale bars are 2 µm.
(PDF)

Figure S7 **Definition of delta values and determining instrumental accuracy.** (A) Equations describing the calculation of delta values, and (B) a standard curve of isotope ratio measurements of *R. palustris* cells grown in known amounts of labeled water showing the precision of the NanoSIMS for ^2^H/^1^H analysis across a large range of ^2^H relative abundance. Error bars represent the standard error for total ions integrated over each cell.
(PDF)

Figure S8 **Visualization of indium time oxide coated slides following NanoSIMS analysis.** (A) SEM image of rastered domains of ion images made of cells of *R. palustris* showing cell material was completed ablated over several frames of NanoSIMS analysis. (B) SEM image of *N. punctiforme* following NanoSIMS analysis shows cells largely intact.
(PDF)

Table S1 Strains plasmids and primers used in this study.
(PDF)
