# Supplementary figures and images for: Probing the Subcellular Localization of Hopanoid Lipids in Bacteria Using NanoSIMS
Source: PLoS One. 2014 Jan 7;9(1):e84455. doi: 10.1371/journal.pone.0084455 (PMC3883690; doi:10.1371/journal.pone.0084455)

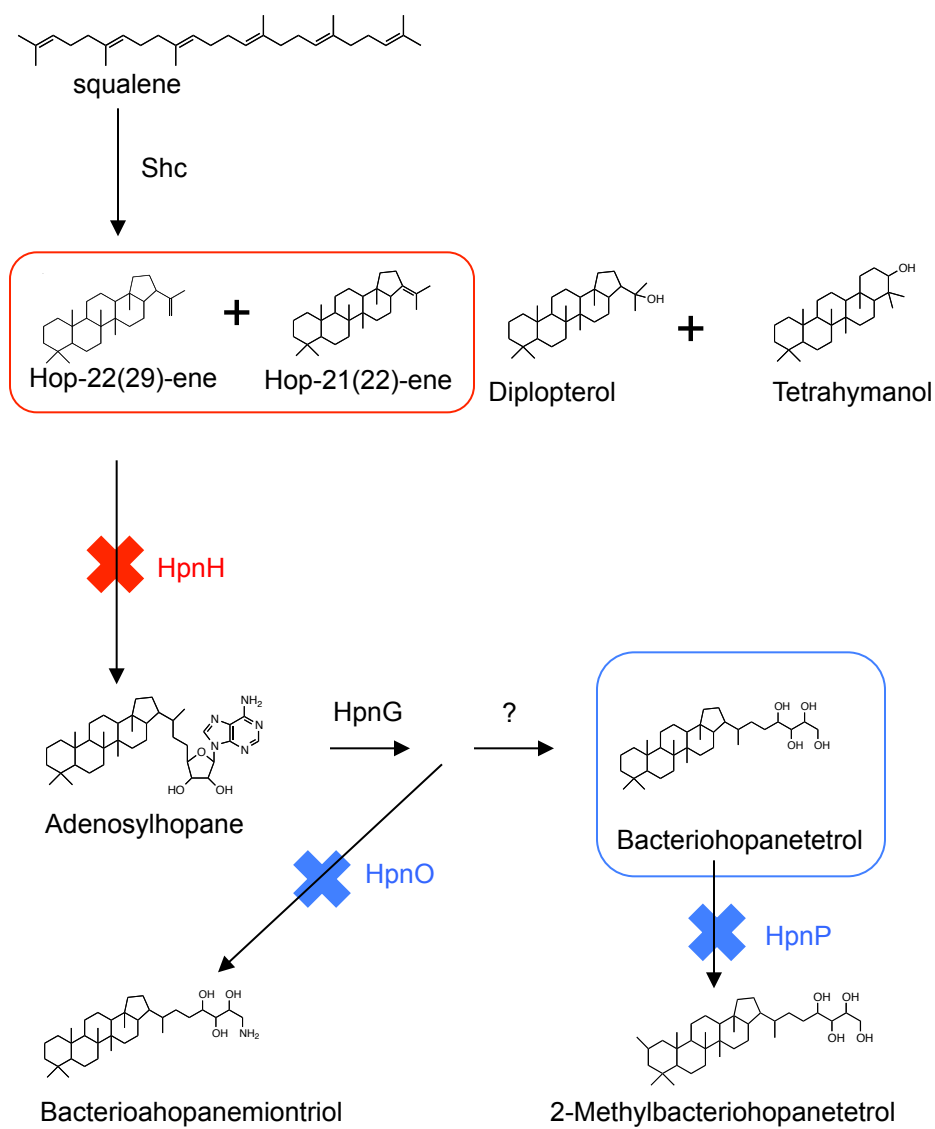

**Figure S1.**

Supplement: Figure S1 — The pathway of hopanoid biosynthesis leading from squalene to the production of hopanoids. Colored X's indicate the disruption of hopanoid biosynthesis through the deletion of known biosynthetic genes. Genes were deleted to control hopanoid production of specific hopanoid lipids and enable the production of unique labeled compounds. Shown in red is the HpnH protein essential for the production of adenosylhopane from Hop-22(29)-ene. Shown in blue are deletions of HpnO, the gene responsible for the production of bacteriohopaneaminotriol, and HpnP, the gene essential to the production of 2-methylbacteriohopanetetrol. (PDF) [file pone.0084455.s001.pdf]

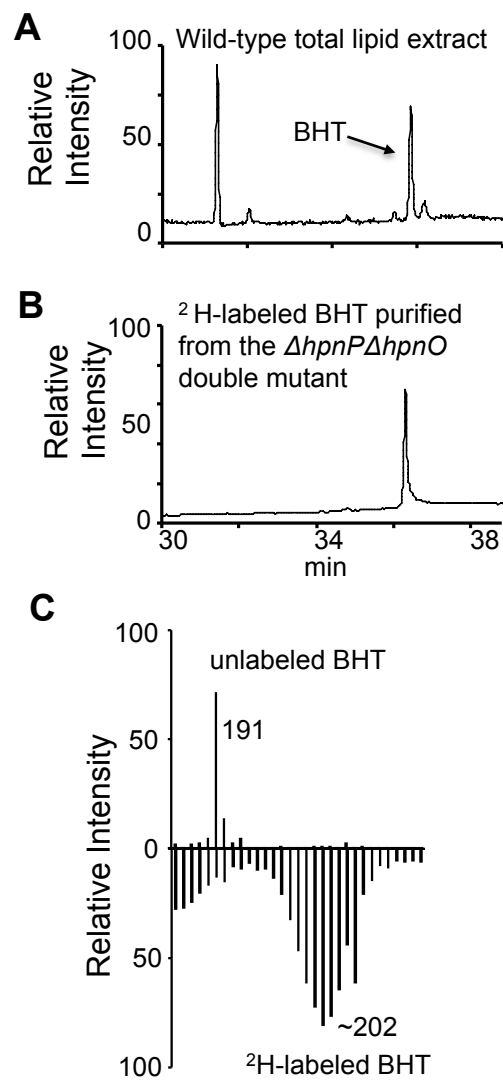

**Figure S2.**

Supplement: Figure S2 — Purification of 2H-labeled BHT. (A) GC/MS chromatogram of total lipid extract of wild-type unlabeled R. palustris shows a range of hopanoid products. (B) GC/MS chromatogram of pure unlabeled BHT. (C) Comparison of the mass spectrums of unlabeled (top) and 2H-labeled (bottom) BHT. The shift of the 191 peak to approximately 202 is consistent with about a 48% non-specific 2H label. (PDF) [file pone.0084455.s002.pdf]

**A**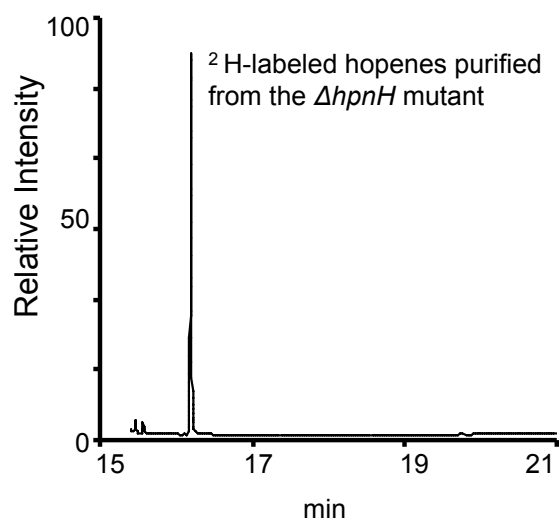**B**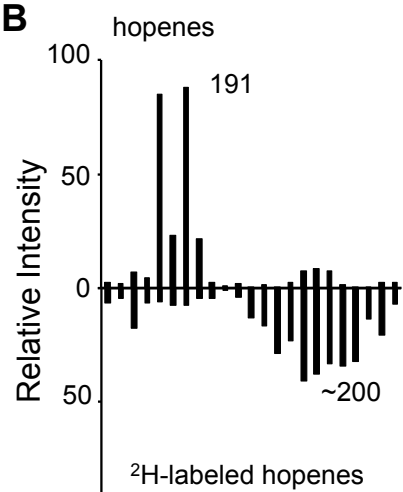**Figure S3.**

Supplement: Figure S3 — Purification of 2H-labeled hopenes. (A) GC/MS chromatogram of purified hopene isomers. (B) Mass spectrum of unlabeled hopene (top) compared to the 2H-labeled spectrum (bottom). The shift of the 191 peak to approximately 200 is consistent with about a 39% non-specific label. (PDF) [file pone.0084455.s003.pdf]

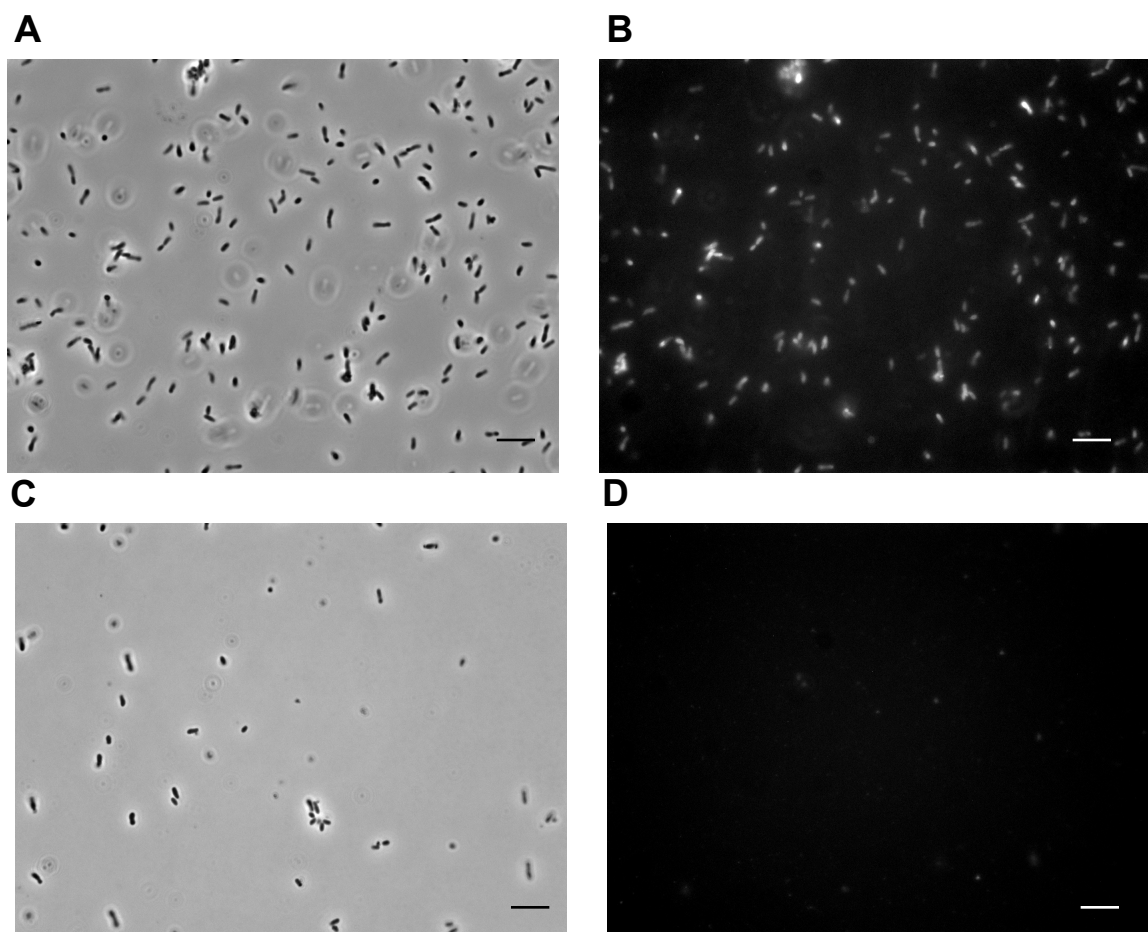

**Figure S4.**

Supplement: Figure S4 — Filipin, a cholesterol specific dye [21] , was used to determine the ability of R. palustris to uptake exogenously added cholesterol. (A) Phase contrast and (B) fluorescence images of R. palustris, stained with filipin, following exposure to cholesterol containing medium. (C) Phase contrast and (D) fluorescence images of R. paustris exposed to medium that did not contain cholesterol. All scale bars are 5 µM. Images are typical of 3 fields of view and 3 biological replicates. (PDF) [file pone.0084455.s004.pdf]

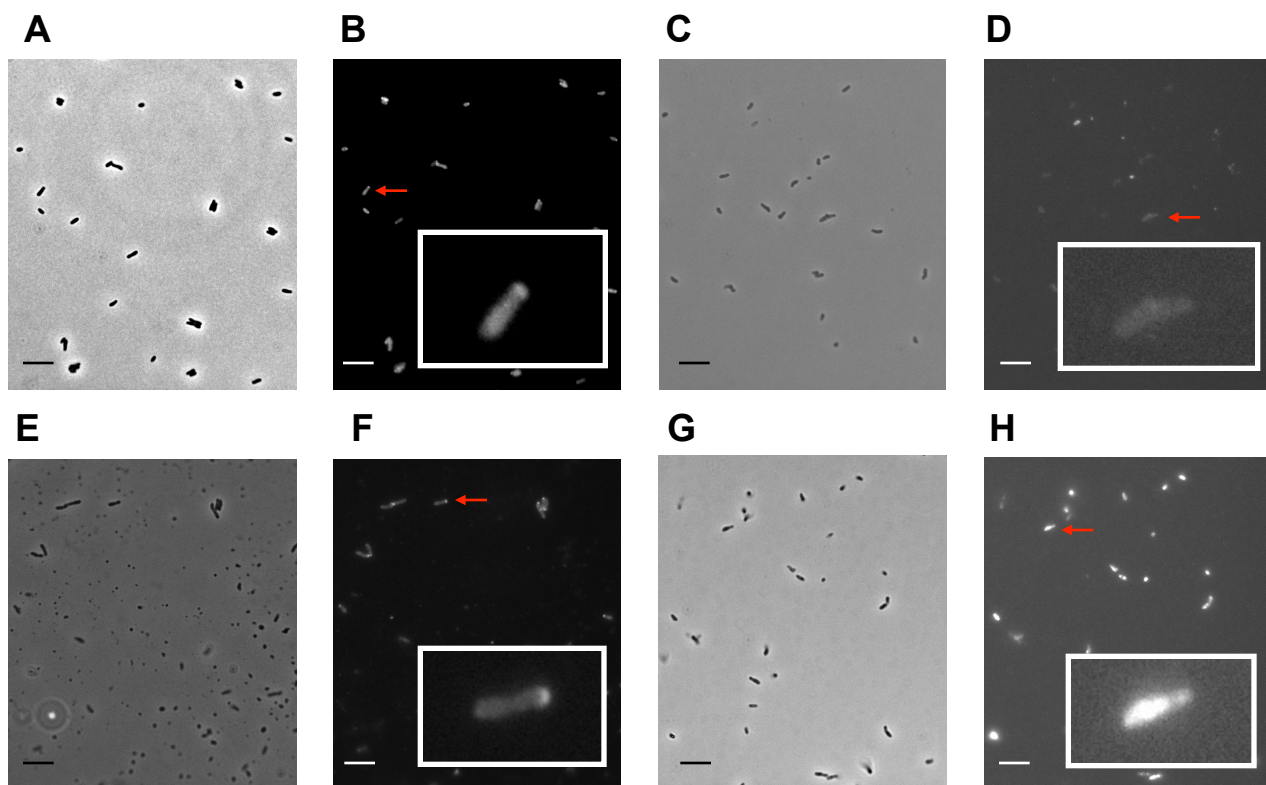

**Figure S5.**

Supplement: Figure S5 — Localization and fluorescence of mCherry labeled Pal in R. palustris . (A) Phase contrast and (B) fluorescence images of the hopanoid-producing R. palustris strain glmX::PlacZ–Pal–mCherry fluoresce following induction by IPTG. (C) Phase contrast and (D) fluorescence images of mCherry Pal in the hopanoid negative mutant Δshc glmX::PlacZ–Pal–mCherry mutant show reduced fluorescence. (E) Phase contrast and (F) fluorescence images show the complementation of the Δshc glmX::PlacZ–Pal–mCherry phenotype 30 min after the the addition of 1 µg/ml of exogenously added hopenes. Yellow arrows in e indicate the presence of small vesicles formed after the addition of the hopene. (G) Phase contrast and (H) fluorescence images showing the complementation of the fluorescent signal of the Δshc glmX::PlacZ–Pal–mCherry phenotype 30 min after the the addition of 1 µg/ml of exogenously added BHT. Cells shown in the insets are indicated by the red arrows. All cultures were induced for 8 hours with 100 µm IPTG. All scale bars are 5 µm. Images are typical of 4 fields of view and 3 biological replicates. (PDF) [file pone.0084455.s005.pdf]

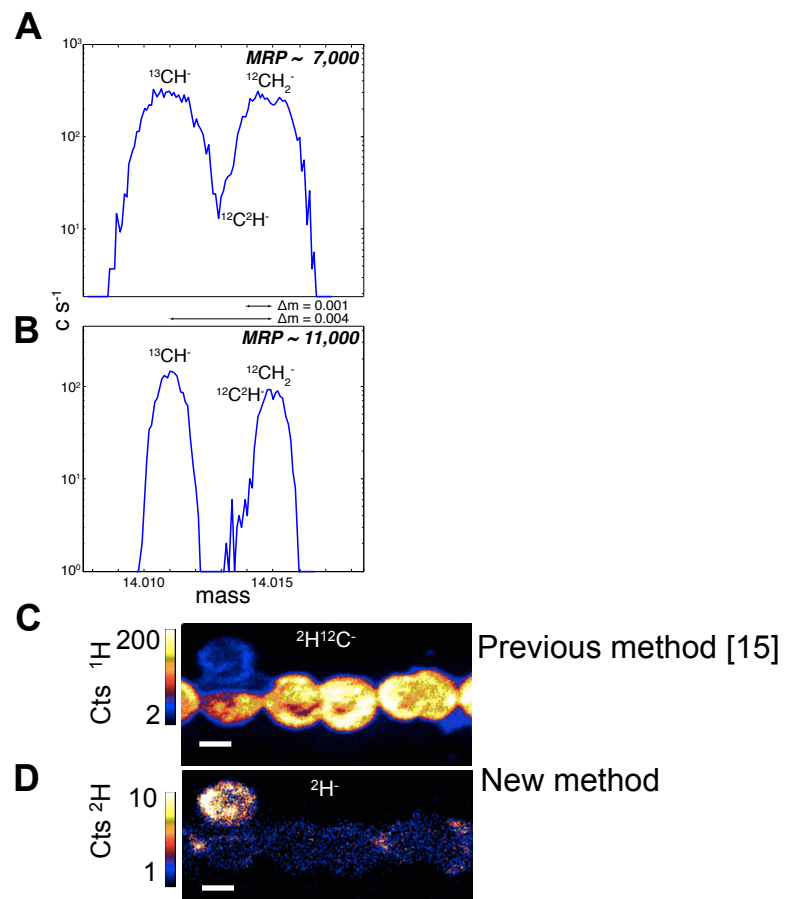

**Figure S6.**

Supplement: Figure S6 — Comparison of methods used for the detection of 2H/1H. Prior methods to measure 2H/1H relied on measurements of the 12C2H− and 12CH− ions [15], the former of which is subject to an isobaric interference from 12CH2 − that precludes this analytical set up for imaging labeled lipids within cells at any reasonable mass resolving power (MRP). (A) Mass spectrum ca. 14 A.M.U. of natural abundance R. palustris cells at the MRP used by Kraft et al. [15]. (B) Same mass spectrum with the highest MRP available at those conditions. Instead the NanoSIMS 50 L enables detection of 1H−, 2H−, 12C−, and 13C− concurrently in the same analysis. Direct comparison of 2H12C− (C) and 2H− (D) yields from the hopene labeled cell material (also shown in Fig. 6). The images in c and d are representative of 5 fields of view and three biological replicates. Scale bars are 2 µm. (PDF) [file pone.0084455.s006.pdf]

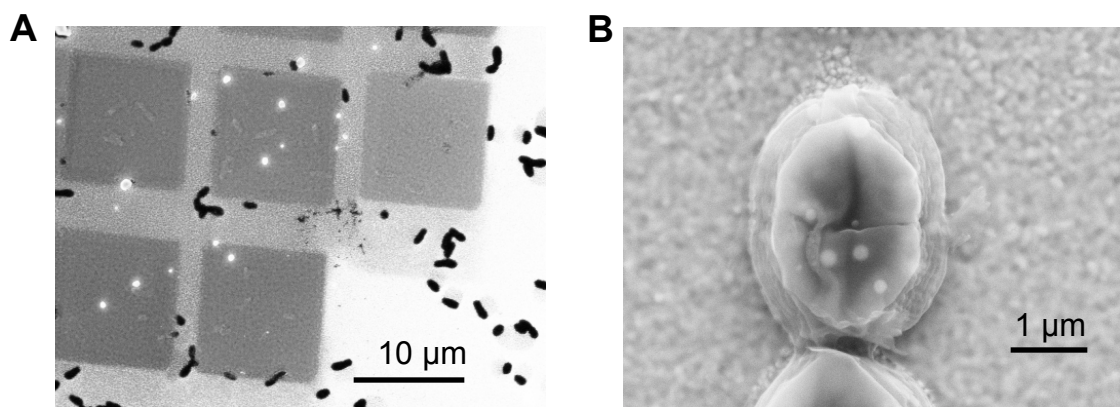

**Figure S8.**

Supplement: Figure S8 — Visualization of indium time oxide coated slides following NanoSIMS analysis. (A) SEM image of rastered domains of ion images made of cells of R. palustris showing cell material was completed ablated over several frames of NanoSIMS analysis. (B) SEM image of N. punctiforme following NanoSIMS analysis shows cells largely intact. (PDF) [file pone.0084455.s008.pdf]
