# Supplementary material for: Probing the Subcellular Localization of Hopanoid Lipids in Bacteria Using NanoSIMS
Source: PLoS One. 2014 Jan 7;9(1):e84455. doi: 10.1371/journal.pone.0084455 (PMC3883690; doi:10.1371/journal.pone.0084455)
Supplement: Figure S7 — Definition of delta values and determining instrumental accuracy. (A) Equations describing the calculation of delta values, and (B) a standard curve of isotope ratio measurements of R. palustris cells grown in known amounts of labeled water showing the precision of the NanoSIMS for 2H/1H analysis across a large range of 2H relative abundance. Error bars represent the standard error for total ions integrated over each cell. (PDF) [file pone.0084455.s007.pdf]

A

$$^{2}H/^{1}H_{\text{natural abundance}} = 0.00015576$$

$$\delta^{2}H = \left(\frac{^{2}H/^{1}H_{\text{sample}} - ^{2}H/^{1}H_{\text{natural abundance}}}{^{2}H/^{1}H_{\text{natural abundance}}} - 1 \right) \times 1000$$

$$^{13}C/^{12}C_{\text{natural abundance}} = 0.0105$$

$$\delta^{13}C = \left(\frac{^{13}C/^{12}C_{\text{sample}} - ^{13}C/^{12}C_{\text{natural abundance}}}{^{13}C/^{12}C_{\text{natural abundance}}} - 1 \right) \times 1000$$

B

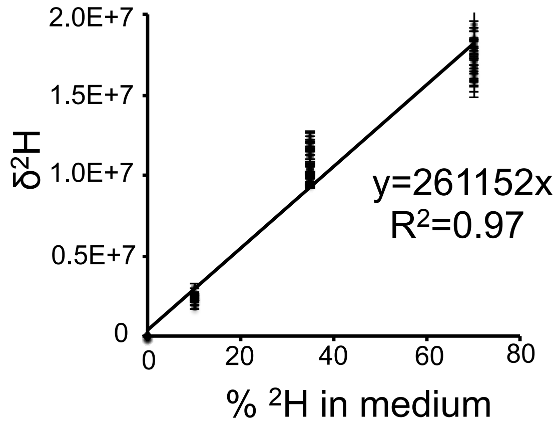

Figure S7.
