# Supplementary material for: Probing the Subcellular Localization of Hopanoid Lipids in Bacteria Using NanoSIMS
Source: PLoS One. 2014 Jan 7;9(1):e84455. doi: 10.1371/journal.pone.0084455 (PMC3883690; doi:10.1371/journal.pone.0084455)
Supplement: Table S1 — Strains plasmids and primers used in this study. (PDF) [file pone.0084455.s009.pdf]

Table S1: Strains plasmids and primers used in this study.

Strains, plasmids, and primers	Genotype, markers, or characteristics	Source
Strains		
R. palustris TIE-1	Wild-type strain	22
R. palustris Δshc	*R. palustris Δshc*	22
R. palustris ΔhpnH	*R. palustris ΔhpnH*	18
R. palustris *ΔhpnG ΔhpnO*	*R.* palustris *ΔhpnG ΔhpnO*	C. H. Wu
glmX::P_{lacZ}—Pal—mCherry	*glmX*::P_{lacZ}—Pal—mCherry	This study
Δshc glmX::P_{lacZ}—Pal—mCherry	*Δshc glmX*::P_{lacZ}—Pal—mCherry	This study
Plasmids		
pGK229	P_{lacI} —LacI—P_{lacZ}—mCherry	This study
pGK234	P_{lacI} —LacI—P_{lacZ}—Pal—mCherry	This study
Primers		
Pal forward	GGCGCGCCCATATGACCAATCACA AGCGAATCC	This study
Pal reverse without stop	TATCTAGAACTAGTGGCGCCGGCG TTCAGCAC	This study
